# Results of the spine-to-rib-cage distraction in the treatment of early onset scoliosis

**DOI:** 10.4103/0019-5413.58602

**Published:** 2010

**Authors:** Marco Teli, Alessio Lovi, Marco Brayda-Bruno

**Affiliations:** Department of Spinal Surgery, Galeazzi Orthopaedic Institute, Milano (Milan), Italy

**Keywords:** Early onset scoliosis, growing rods, spinal deformity

## Abstract

**Background:**

Growing rod systems have been used in the last 30 years for the treatment of early onset scoliosis (EOS) with variable success rates. We report the results of treatment of EOS with a newly developed hybrid rod distraction system applied to the rib cage and spine with a nonfusion technique in a prospective multicenter clinical trial.

**Materials and Methods:**

A total of 22 patients affected by progressive EOS resistant to cast and/or brace treatment were enrolled from 2004 to 2005 after informed consent into a trial of surgical treatment with a single spine-to-rib growing rod instrumentation growing spine profiler (GSP). Curves >60° Cobb in the frontal plane or bending < 50% were addressed with staged anterior annulotomy and fusion and posterior implantation of a GSP rod. Less severe and rigid curves were treated with posterior implantation of GSP only. The elongation of GSP was planned according to spinal growth. Patients were kept in a brace between elongations.

**Results:**

A total of 20 patients were available to follow-up with complete data. The mean follow up is 4.1 years. Mean age at time of initial surgery was 5 years (3–8). Nine patients had staged antero-posterior surgeries, 11 posterior only surgeries. Mean spinal growth was 1.9 cm (1.5–2.3) or 0.5 cm per year. Mean coronal Cobb's angle correction was from 56° to 45°. Major complications affected 40% of patients and included rod failure in 6/20 and crankshaft in 5/20 (all in the anteroposterior surgery group).

**Conclusion:**

Treatment of EOS with spine-to-rib growing rod in the present form provides similar correction and complication rates to those published in the series considering traditional single or dual growing rod systems. Based on this, the authors recommend revision of the GSP design and a new clinical trial to test safety and efficacy.

## INTRODUCTION

Early onset scoliosis (EOS) is a deformity of the growing spine that affects children before the age of complete lung maturation, i.e., 8–10 years.^1^ Growing children with progressive spinal deformity resistant to casting and/or bracing have been treated for decades with “spinal instrumentation without fusion” or growing rods.^2^,^3^ The terms encompass a range of posterior spinal instrumentation techniques—namely single or dual growing rods and expandable ribs—having the common goal of obtaining progressive deformity correction without halting the growth of the spine and lungs.^3^ Traditional single growing rods have a reported incidence of rod breakage and deep infection of 42% and 9%, respectively.^4^ Dual growing rods implanted subfascially have a reported 22% rate of implant failure and a 9% rate of deep infection.^2^ The recent introduction of rib instrumentation has triggered new enthusiasm for the possibility of treating both EOS and complex congenital deformities^1^,^3^,^5^ indirectly by acting on the chest wall rather than on the spine itself.

It was therefore conceived that a spinal instrumentation able to join the features of rib distraction with those of spinal distraction would take advantage of both techniques by avoiding violation of the most part of the growing spine during treatment of EOS. We report the results of treatment of EOS with a newly developed hybrid rod distraction system applied to the rib cage and spine with a nonfusion technique in a prospective multicenter clinical trial.

## MATERIALS AND METHODS

**Figure 1:**
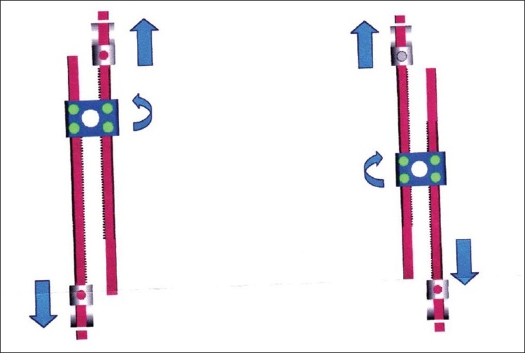
Implant scheme: GSP composed of two 3.5mm rods and a central connector hosting a gear for distraction. Clockwise rotation of the gear provides distraction of the rods i.e. lenghtening of the implant.

**Figure 2:**
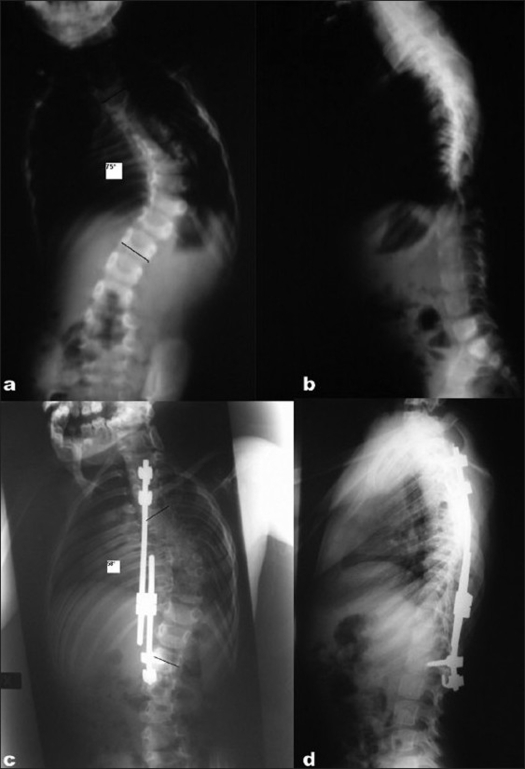
Preoperative anteroposterior (a) and lateral (b) views of a 2 yr old patient with early onset scoliosis. Anteroposterior (c) and lateral (d) views of the same patient treated with GSP at the age of 4 yrs depicting a correction fo 17° in Cobb's angles with continued spinal growth

22 children affected by progressive early onset scoliosis resistant to conservative treatment (serial casting or bracing) underwent surgical implantation of a single spine-to-ribs growing rod [Figures 1 and 2] from 2004 to 2006. The etiology of EOS was divided as follows: 10 cases were idiopathic, 5 congenital, 3 neuromuscular, 1 neuropathic and 1 due to arthrogryposis. Surgeries were performed at five different European centers for spinal diseases by an equivalent number of spinal deformity surgeons. All patients were skeletally immature at the time of surgery as demonstrated by spinal posteroanterior X-rays (absence of ossification of both the iliac apophyses and the triradiate cartilages).

### Operative procedure

Prior to the present surgery, no patient had been operated on for his or her deformity. In the study group, patients with curves > 60° Cobb in the frontal plane or bending < 50% were addressed with staged anterior annulotomy and fusion 1 week prior to implantation of a growing spine profiler (GSP) rod. Less severe and rigid curves were treated with posterior implantation of GSP only.^3^,^4^ Anterior fusion is a growth arrest procedure traditionally aiming at preventing the occurrence of continuous anterior growth and “crankshaft” worsening deformity when serious EOS is only operated posteriorly.^2^–^4^,^6^ Anterior fusion is performed through a thoracotomy or a thoracoabdominal approach depending on the level of maximum spinal deformity to be addressed.

GSP rods (Paradygm Spine, Paris France) are stainless steel 3.5 mm diameter rods connected by a central connector hosting a gear that allows distraction, i.e., elongation of the rods when rotated clockwise and compression when rotated anticlockwise [Figure 1]. In this study, GSP rods were used to control and distract spinal deformities through a spine-to-rib design. GSP rods are currently also used as rib-to-rib chest wall expanders in a different multicenter trial. For the implantation of GSP, the upper rib and lower end vertebra were exposed through a posterior midline approach. Rib hooks and laminar hooks or pedicle screws were applied with sizes appropriate to the surgical anatomy of the patient. Diameter pedicle screws, 4.5 mm, were applied to the lumbar spine. Only the bottom end vertebra was decorticated and no bone graft was applied *in situ* to promote local arthrodesis. Two rods were then contoured for sagittal balance, connected to the hooks and/or screws and implanted in a subfascial position on the concavity of the coronal curvature as in previously designed spinal systems.^4^,^7^ At 10-s time interval every 2 mm of distraction was observed in order to allow for the viscoelastic properties of the spinal soft tissues to act.^8^,^9^ The distraction normally stopped after 15–20 mm of elongation and in any case until the implant demonstrated inability to sustain further manual elongation without undue signs of rotation or dislocation. This is customary when growing rods of any modern kind are applied.^2^

### Protocol of management

Once patients had undergone the first implantation and distraction, they were kept on Milwaukee spinal braces until the following distraction that was scheduled according to the rate of their spinal growth. This on average happens every 6–9 months in the age group from 5 to 10 years.^2^,^3^ For every subsequent distraction surgery, only the rod connector and 50mm of each rod were exposed through a centered skin incision and opening of the fascia. Loosening of the connector locking nuts and rotation of the gear followed this. The elongation of the rods stopped when the implant was unable to sustain further manual elongation without signs of rotation or instability, as in the case of the first implantation and distraction.

These distraction surgeries were repeated until the child reached the maximum predicted spinal growth as indicated by the progressive ossification of the iliac apophyses and triradiate cartilages, i.e., normally around puberty.^3^,^4^ Between elongations, patients were prescribed the use of a molded spinal Milwakee-type brace to protect the instrumentation from undue force vectors. Patients who reached puberty after undergoing serial distraction of growing rods were scheduled for a definitive posterior spinal fusion and removal of growing rods.^2^,^3^

## RESULTS

A total of 20 out of 22 (91%) treated patients completed the prospective 2-year minimum follow-up to be included in the analysis of data. The mean follow-up from the index surgery was 4.1 ± 1.5 (range, 2–5.4) years. The sample group consisted of 8 males and 12 females aged on average 5.3 ± 1.7 (range, 3.2–8.5) years at the time of surgery. Nine patients had an anterior growth arrest surgery prior to the implantation of growing rods because of curves > 60° Cobb in the frontal plane or bending < 50%. After 1 week, these patients underwent an implantation of posterior GSP growing rods. Eleven patients underwent only posterior surgery in the form of implantation of GSP growing rods. Subsequent serial distractions of growing rods were then performed according to the protocol described above. The average number of distraction surgeries was 3.5 ± 1.1 (range, 2.3–4.4) per case until final follow-up, with an average time interval between distractions of 7.2 ± 2.5 (range, 5.7–10.4) months. Table 1 displays features of pre- and follow-up spinal deformities. Mean coronal Cobb's angle correction was from 56° to 45°. With an average of less than 2-cm rod distraction per single surgery, minimal corrections were obtained in Cobb's angles indicating that distractions re-tensioned implants^3^,^4^ but allowed a mean spinal growth (SG) of 1.9 ± 0.4 (1.5–2.3) cm [Table 2]. Major complications affected 8 out of 20 patients (40%) and included rod failure in 6/20 (30%) and crankshaft in 5/20 (25%). Surprisingly, all of these crankshaft cases were in the anteroposterior surgery group. These five patients underwent elective removal of growing rods and posterior instrumented fusion to control the newly developing deformity. In one of these five patients, two debridements of the surgical wound were necessary to control the development of a deep infection sustained by Staphylococcus epidermidis. There were no instances of fracture of the posterior vertebral elements during or after distraction. The six cases of implant failure were divided as follows: two cases of rod breakage requiring revision to higher diameter traditional growing rod systems, one case of rib--hook cut-out (failure of the bone-hook interface) requiring repositioning of the hook to a different rib, and three cases of loosening of lumbar pedicle screws requiring revision to a different level or to laminar hooks. Deep infections affected 2 out of 20 (10%) cases (including the one described above) and were resolved with repeat debridement and targeted i.v. antibiotic treatment.

**Table 1:** Spinal deformity angles and lengthening data

	Pretreatment PA Cobb's angle (°)	Follow-up PA Cobb's angle (°)	Pretreatment lat. Cobb's angle (°)	Follow-up lat. Cobb's angle (°)	Total rod distraction (mm)
Mean	56.2 (main curve)	45.2 (main curve)	46.2	41.8	19.2
SD	19.9	20.0	13.7	13.5	4.4
Range	40–78	25–65	33–69	29–55	15–24
*P* (*t*-test)		0.003		0.006	

**Table 2:** Spinal growth

	Pretreatment sitting height (cm)	Follow-up sitting height (cm)	Spinal growth (cm)
Mean	66.2	68.1	1.9
SD	4.5	4.3	0.2
Range	61.5–71	64–74	1.5–2.2
*P* (*t*-test)			0.07

Figure 2 shows the follow-up at 2 years of a typically severe EOS case with spinal imbalance on the coronal plane with good sagittal balance. At present follow-up, three cases underwent definitive posterior spinal fusion without complications. In all of these cases, direct inspection of the operated sites demonstrated bony fusion at the spinal instrumented level and at the one immediately above, and the formation of abundant new bone (hyperostosis) around the rib hooks.

## DISCUSSION

Infection and implant failure are the two main limits of the current growing rod techniques.^2^,^4^,^6^,^7^,^10^ In this study, fair control of the spinal deformity along with continuing spinal growth (1.9 cm in four years equivalent to 0.5 cm per year) was obtained. Spinal growth was in the lower range of previously published material on the issue of EOS (ranging from 2 to 4.8 cm overall). Complications were too frequent and serious to judge results in a positive way despite a lower infection rate (10%). Particularly, rod failure (25%) and crankshaft phenomenon (30%) deserve comments and comparison with similar series. Implant failure, seemingly a function of the length of treatment with growing rods, is usually due to implant loosening or rod breakage.^4^,^6^,^7^,^10^ Possibly, this problem could be addressed by distracting the growing rod system nonsurgically at closer intervals than the 6 to 9 month routine^11^ or by using bigger diameters for the manufacturing of the rods.^3^ The crankshaft phenomenon is dependent on the age and skeletal maturity of the patients at the time of spinal fusion, as well as on the seriousness of spinal deformity itself.^2^–^4^ As stated above, scoliotic deformities with a Cobb's angle above 60° and bending below 50% were all addressed with anterior annulotomy and growth arrest prior to the implantation of growing rods. Nevertheless, this approach did not stop 30% of our patients from developing increasing deformity in the crankshaft fashion. All previous authors have reported similar problems with single growing rods. Paul Harrington in 1962 first described the use of a single, threaded growing rod on the concave side of the deformity, reporting poor results due to spontaneous fusion and a 11% incidence of rod failure.^10^ Moe *et al* developed the use of a subcutaneous growing rod in an attempt to limit the incidence of implant failure and infection. They achieved good mean curvature control and 3.8 cm mean SG at follow-up, at the expense of a 50% rod failure, 20% crankshaft, and 15% infection rate.^6^ Klemme *et al* reported on the use of a subfascial rod and achieved 3.1 cm mean SG, with an 8% rod failure and a 15% infection rate.^7^ Mineiro *et al*, reported on subcutaneous rodding (with or without anterior apical fusion) and achieved 2.0 cm mean SG, with 42% rod failure, 25% crankshaft and 9% infection rates.^4^ Akbarnia *et al* reported on the use of two parallel growing rods implanted subfascially with a connector for periodic lengthening, achieving 4.6 cm mean SG, with a 22% rate of implant failure, 9% rate of deep infection and only 4% crankshaft, justified by the authors with the use of dual parallel rods.^2^

In the present series, the high incidence of rod failure and crankshaft required unplanned surgery and added morbidity to the treatment. Spinal growth was in the lower range compared to similar series. The infection rate was not worrying. These figures were obtained within a protocol of treatment of EOS that was largely accepted at the time of the start of the study^2^–^4^—anterior fusion when needed, close monitoring of spinal growth, distractions every 6–9 months, use of braces between distractions—with the single variable of the newly designed rods. We must therefore conclude that treatment of EOS with a single spine-to-rib growing rod (GSP) in the present form requires revision of the design and application of the implant with a new clinical trial to test safety and efficacy.
